# Supplementary material for: The Relationship between Concentration Effort, Focus Back Effort, Focus Back State, and Mind Wandering
Source: Behav Sci (Basel). 2024 Feb 22;14(3):162. doi: 10.3390/bs14030162 (PMC10967797; doi:10.3390/bs14030162)
Supplement: Supplementary file 1 [file behavsci-14-00162-s001.zip › behavsci-2824170-supplementary.pdf]

Supplementary Table S1. Gender difference between variables of interest.

Measure	*t*	*df*	*p*
CE	1.66	119	0.100
FBE	0.43	119	0.666
FBS	0.37	119	0.712
MW	0.15	119	0.884
Omission errors	-0.97	119	0.336

Note. CE = concentration effort; FBE = focus back effort; FBS = focus back state; MW = mind wandering.

Supplementary Table S2. Partial correlation coefficients for all primary variables of interest.

Measure	CE	FBE	FBS	MW	Omission errors
CE	-	0.76***	0.54***	-0.49***	-0.15
FBE		-	0.79***	-0.43***	-0.01
FBS			-	-0.26**	0.06
MW				-	0.29**
Omission errors					-

Note. CE = concentration effort; FBE = focus back effort; FBS = focus back state; MW = mind wandering. ***$p < 0.001$, **$p < 0.01$.
